# Reproductive tract microbiome dysbiosis associated with gynecological diseases

**DOI:** 10.3389/fcimb.2025.1519690

**Published:** 2025-02-18

**Authors:** Qingyue Zheng, Tianshu Sun, Xiaochuan Li, Lan Zhu

**Affiliations:** ^1^ National Clinical Research Center for Obstetric & Gynecologic Diseases, Beijing, China; ^2^ Department of Obstetrics and Gynecology, Peking Union Medical College Hospital, Chinese Academy of Medical Sciences & Peking Union Medical College, Beijing, China; ^3^ Clinical Biobank, Medical Research Center, National Science and Technology Key Infrastructure on Translational Medicine, Peking Union Medical College Hospital, Chinese Academy of Medical Sciences and Peking Union Medical College, Beijing, China

**Keywords:** reproductive tract, microbiome, gynecological diseases, vaginal microbiome, endometrial microbiome, endometrial cancer, endometriosis (EM)

## Abstract

Female health and the microbiota of the reproductive tract are closely associated. The research scope on reproductive tract microbiota extends from the vaginal to the upper reproductive tract and from infectious diseases to various benign and malignant gynecological and obstetrical diseases. The primary focus of this paper was to evaluate the most recent findings about the role of reproductive tract microbiota in gynecological diseases, including endometrial polyps, uterine fibroids, endometriosis, adenomyosis, endometrial hyperplasia, and endometrial carcinoma. Different stages of gynecological diseases have diverse microbiota in the female reproductive tract, and some specific bacteria may help the disease progress. For example, *Fusobacterium* may exacerbate endometriosis, while treatments that target microbiota, such as antibiotics, probiotics, and flora transplantation, showed some efficacy in the experiment. These findings indicate the wonderful prospect of this field. Additionally, we have discussed how microbiome research can improve our understanding of the interactions between reproductive tract microorganisms and hosts, aid in the screening and diagnosis of gynecological diseases, and direct the development of preventive and therapeutic strategies aimed at maintaining and restoring a healthy reproductive tract microbiota when combined with other technologies like transcriptome and proteome, *in vitro* cultured cells, and animal models.

## 1 Introduction

Research on the microbiota of the female reproductive system was mainly focused on the lower reproductive tract in the era when technology based on culture was used to detect the composition of microorganisms (Medina-Bastidas et al., 2022). *Lactobacillus* makes up most of the vaginal microbiota in healthy women of childbearing age, which prevents the growth of pathogenic microorganisms by maintaining a low vaginal pH, competing for nutrients, preventing adhesion, producing antimicrobial metabolites, and regulating the local immune response (Verstraelen et al., 2022). During this time, the research on the connection between vaginal microbiota and health was mainly focused on a few prevalent infectious diseases, such as vulvovaginal candidiasis and bacterial vaginosis (Pereira et al., 2021).

Two innovation points in the research of reproductive tract microbiota emerged with the advancement of genomics, particularly meta-genomics and next-generation sequencing (NGS) technologies (Li et al., 2018). First, researchers became aware of the microbiota in the upper reproductive tract, which was previously believed to be sterile. According to studies, the uterus of healthy people is microbially colonized, and the health of the female reproductive system is directly correlated with the microbiota of the upper reproductive tract (Rampersaud et al., 2012). Due to ethical considerations and limitations in sampling methods (Yuan et al., 2024), studies on the endometrial microbiome of healthy individuals without any gynecological or obstetric disorders are indeed scarce. Although there is still debate on the makeup of a healthy endometrial microbiota, mounting research suggests that *Lactobacillus* is the most prevalent genus in a relatively healthy uterus (Chen et al., 2017; de Medeiros Garcia Torres and Lanza, 2024; Peric et al., 2019). In addition, the focus of study on reproductive tract microbiota has shifted from infectious diseases to a range of obstetrical and gynecological diseases, including endometrial cancer, endometriosis, and infertility. These discoveries might help with the pathogenesis of the disease, diagnosis, and treatment. This paper primarily evaluated recent developments in the research of reproductive tract microbiota in gynecological diseases and examined the relationship and potential interactions between microbiota and common gynecological diseases. It also discussed the possibility of using the microbiome as an adjunct to current treatments and a potential biomarker for identifying gynecological diseases.

## 2 Reproductive tract microbiota in gynecological diseases

### 2.1 Endometrial polyps

Endometrial polyps (EP), a prevalent benign gynecological disease, with an incidence of 7.8%-34.9% (Anonymous, 2012; Salim et al., 2011), is characterized by excessive growth of glands, stromal cells and blood vessels in the endometrium, which is usually associated with abnormal uterine bleeding (AUB). According to some studies, EP is associated with chronic endometritis (CE) (El-Hamarneh et al., 2013), whereas CE may result from an overgrowth of *Ureaplasma urealyticum* or common bacteria (Cicinelli et al., 2009). Thus, the EP may be related to the prolonged existence of certain microorganisms. The vaginal and uterine microbiomes of patients with EP, patients with EP and CE, and healthy women were compared in a 2016 study with 10 subjects in each group. It was discovered that while *Proteobacteria*, *Firmicutes*, and *Actinobacteria* dominated the intrauterine microbiota of all samples, the proportion of *Proteobacteria* was much lower in patients with EP than that of healthy women, while the proportion of *Firmicutes* was significantly higher than that of healthy women. In addition, the proportion of *Pseudomonas* was significantly lower in patients with EP, while the proportion of *Lactobacillus*, *Gardnerella*, *Bifidobacterium*, *Streptococcus*, and *Alteromonas* was significantly higher than the control group. Patients with EP generally had a more diverse intrauterine microbiome than the control group. Whether EP patients were with CE or not had no significant effect on their intrauterine microbiome (Fang et al., 2016). It is worth noting that this study included healthy women as the control group, which few other studies did. These women underwent hysteroscopy and laparoscopy because of infertility of their male partners, while they had regular menstrual cycles and no structural abnormalities of the uterus. This eliminates the interference, making it possible to figure out the characteristic bacteria of the disease.

Liang et al. prospectively collected vaginal, cervical and uterine microbiological samples from 134 infertile patients. They found that the microbiota in the reproductive tracts of patients with CE and EP differed from that of the healthy control group. For example, compared with the control group, the distribution of *Firmicutes* throughout the reproductive tract of EP patients was significantly increased. In contrast, the distribution of *Proteobacteria* was significantly lower, which is consistent with the findings of previous studies. In contrast to previous studies, the abundance of *Lactobacillus* in the EP group was lower than that in the control group (Liang et al., 2023). Their study was an essential reference for subsequent research because it included a sufficient sample size and adequate sampling locations. Future research on the microbiome of endometrial polyps may focus on specific clinical problems, such as recurrence and response to treatment, to identify suitable biomarkers or therapeutic targets.

### 2.2 Leiomyoma

Leiomyomas, or uterine fibroids, are the most prevalent benign gynecological neoplasms. They are characterized by muscle and fibrous tissue growths that can cause infertility, heavy or prolonged menstrual bleeding, anemia (Donnez and Dolmans, 2016; Zepiridis et al., 2016), and significant personal and social problems (Millien et al., 2021). There is an increasing understanding of hormones, genetics, epigenetics, and growth factors in the development and progression of uterine fibroids (Torres-de-la-Roche et al., 2017); however, the exact pathogenesis is still unclear. According to some research, bacteria may cause inflammation that contributes to the pathogenesis of uterine fibroids. A study reported that the TLR4/MyD88/NFKB signaling pathway in primary cultured human fibroblasts from leiomyomas was activated under *E. coli* LPS treatment, which suggested that bacteria may be involved in the pathogenesis of leiomyomas by inducing cell proliferation through inflammation (Guo et al., 2015).

According to a study comparing the microbiota of the vagina, cervix, endometrium, and pouch of Douglas in 20 patients with leiomyoma and controls, *Lactobacillus* sp. was found to be less abundant in the vaginal and cervical samples from leiomyoma patients, but *L. iners* was more abundant in the cervix (Chen et al., 2017). There was no significant difference in microbial diversity between patients with leiomyoma and controls in another study that examined the vaginal and cervical microbiomes of 29 patients with leiomyoma and 38 healthy women. Interestingly, alpha diversity decreased in patients with leiomyoma as the number of fibroids increased. At the phylum level, an increase in *Firmicutes* in the vagina and cervix was observed in patients with leiomyoma. *Erysipelotrichaceae* UCG-003 and *Sporolactobacillus* were significantly less common in patients with leiomyoma, whereas *Erysipelatoclostridium*, *Mucispirillum*, and *Finegoldia* were significantly enriched in the differential analysis of relative abundance. Moreover, the microbial co-occurrence networks exhibiting lower connectivity and complexity in patients with leiomyoma suggested decreased interactions and stability of the microbiota in these patients compared to healthy individuals (Mao et al., 2023). The research mentioned above revealed the characteristic microbiome of the reproductive tract associated with leiomyoma, provided clear evidence of microbial dysbiosis in patients with leiomyoma, and restricted the scope of screening for associated microorganisms potentially implicated in the pathogenesis of leiomyoma. More research is required to fill the knowledge gap of microbial dysbiosis and leiomyoma interaction.

### 2.3 Endometriosis

Endometriosis, a gynecological disease that affects 6%-10% of women of childbearing age worldwide, is characterized by the growth of endometrial tissue outside the uterus, including glands and stroma. This disease results in progressive secondary dysmenorrhea and difficulty in having sexual intercourse as well as infertility (Dai et al., 2018). The pathogenesis of endometriosis has not been clarified yet, and scientists are primarily interested in the retrograde menstruation hypothesis. However, 90% of the women have retrograde menstruation, while only 10% have the disease (Akiyama et al., 2019; Bellelis et al., 2019; Gueuvoghlanian-Silva et al., 2018; Khan et al., 2018; Zhang et al., 2018), indicating that other factors may play a role in the pathogenesis of endometriosis. Research has demonstrated that the abundance of bacterial colonization in menstrual blood and endometrial tissue of patients with endometriosis is higher than that of average female (Khan et al., 2018, 2016). Therefore, it is essential to investigate if the microbiome of the reproductive tract is involved in the pathogenesis of endometriosis.

There is more clinical research on endometriosis and microbiome than on uterine fibroids and endometrial polyps (Akiyama et al., 2019; Ata et al., 2019; Chang et al., 2022; Chao et al., 2021; Chen et al., 2020; Hernandes et al., 2020; Huang et al., 2021; Le et al., 2022; Lu et al., 2022; Oishi et al., 2022; Perrotta et al., 2020; Wei et al., 2020; Wessels et al., 2021). In terms of vaginal microbiome, it was discovered that *Lactobacillus* was less common in the vaginal microbiome of patients with endometriosis than in the control group (Chao et al., 2021; Findeklee et al., 2023; Hernandes et al., 2020; Lu et al., 2022). The vaginal microbiome of patients with stages III or IV endometriosis and healthy women were compared by Ata et al (Ata et al., 2019). Patients with endometriosis did not have any detections of *Gemella* and *Atopobium* spp. Another research studied community state type (CST) in greater detail (Perrotta et al., 2020). They used machine learning methods to construct a classification model based on random forest to predict the Revised American Society for Reproductive Medicine (r-ASRM) stage of endometriosis. The study discovered that the *Anaerococcus* from the *Firmicutes* phylum could predict whether patients had stage I-II or III-IV endometriosis after accounting for the menstrual cycle. As the vaginal sample is convenient to obtain, the above research findings have great clinical application potential.

In terms of cervical microbiome, Akiyama et al. demonstrated that compared with the control group, patients with endometriosis had higher levels of *Corynebacterium*, *Enterobactericaea*, *Flavobacterium*, *Pseudomonas*, and *Streptococcus* in their cervical microbiota (Akiyama et al., 2019). NGS results were further confirmed using a real-time polymerase chain reaction to quantify bacteria. They found a statistically significant difference in the abundance of *Enterobacteriaceae* and *Streptococcus* between endometriosis and control. Furthermore, patients with stage I-II endometriosis and those with stage III-IV endometriosis exhibit differences in cervical microbiomes. For stages I and II, the researchers suggested *L. jensenii* or members of the *Corynbacteriales*, *Porphyromonadaceae*, and *Ruminococcaceae* as potential microbial biomarkers; for stages III and IV, they suggested *Bifidobacterium breve* and members of *Streptococcaceae* (Chang et al., 2022). Studies on vaginal and cervical microbiota of patients with endometriosis have shown an increase in the abundance of some low-abundance genera, including *Fannyhessea*, *Prevotella*, *Streptococcus*, *Bifidobacterium*, and *Veillonella*. The degree of endometriosis is correlated with the ratio of the cervical to vaginal abundance of these genera (Yang et al., 2023), indicating that specific genera may migrate between the vagina and cervix and contribute to the pathogenesis of endometriosis.

In the aspect of endometrial microbiome, a study included 12 patients with endometriosis and 9 subjects without endometriosis. It was found that among individuals with endometriosis, the abundance of the *Tepidimonas* genus and *Oxalobactaceae* and *Streptococcaceae* families increased, whereas the *Ralstonia* genus and *Burkholderiaceae* family decreased (Wessels et al., 2021). Another study included 36 patients with endometriosis and 14 control subjects and discovered a significant increase in *Pseudomonadaceae* in endometriosis. Notably, there was a significant increase in *Sphingobium* sp. and *Pseudomonas viridiflava* in the endometrium and peritoneal fluid in endometriosis (Wei et al., 2020), indicating that these could be potential biomarkers.

According to some researchers, an imbalance in microbiota may disrupt the immune system, which could increase pro-inflammatory cytokines, disrupt immune surveillance, and change the profiles of immune cells. This immune disorder could become chronic inflammation, creating a vicious cycle leading to endometriosis (Jiang et al., 2021). Thus, research is being done on how to treat endometriosis by regulating the balance of microbiota. Patients with endometriosis had a significantly higher positive *Fusobacterium* rate in their endometrium than the control group. Following *Fusobacterium* infection of endometrial cells, the transforming growth factor-β signal pathway was activated, resulting in the transformation of quiescent fibroblasts into transgelin-positive myofibroblasts, which obtained the ability of adhesion, migration and proliferation *in vitro*. In the mouse model, the inoculation of *Fusobacterium* increased the number and weight of lesions of endometriosis, while therapy with metronidazole and chloramphenicol decreased the lesion. These results showed that endometriosis pathogenesis might involve *Fusobacterium* infection (Muraoka et al., 2023). Another study using animal models showed that the use of antibiotics or vaginal flora transplants in the vagina effectively treated endometriosis in mice (Lu et al., 2022). In this study, endometriosis mouse models were first established, then 10 mice received vaginal microbiota transplants from healthy mice, and 10 served as controls. The group receiving the vaginal microbiota transplant exhibited smaller endometriotic lesions compared to the control group (p < 0.05). The expression levels of cell proliferation marker Ki-67 and macrophage marker Iba-1 were both reduced in the transplant group. In terms of inflammatory cytokines, the levels of IL-1β, IL-6, and TNF-α in the peritoneal fluid of the microbiota-transplanted mice were lower than those in the control group. Additionally, the expression of TLR4, MyD88, and p65/p-p65 (NF-κB signaling pathway) in the lesions of the microbiota-transplanted mice was downregulated. Taken together, these results suggest that vaginal microbiota transplantation may modulate immune responses by regulating signaling pathways such as NF-κB and influencing the levels of inflammatory cytokines, thereby inhibiting the progression of endometriosis. Furthermore, this study implies that reproductive tract microbiota transplantation may have potential therapeutic effects not only for infections such as bacterial vaginosis but also for gynecological disorders like endometriosis. In patients with stage III and IV endometriosis, oral *Lactobacillus gasseri* OLL2809 relieved pain, according to a placebo-controlled randomized clinical trial (Khan et al., 2018). Another study discovered oral levofloxacin decreased cell proliferation, inflammation, and angiogenesis in endometrium and endometriosis lesions (Khan et al., 2021). The abovementioned studies demonstrated the promising future of the therapy targeted dysbacteriosis in treating endometriosis.

### 2.4 Adenomyosis

Adenomyosis is a benign disease characterized by the invasion of endometrial glands and stroma into the myometrium, resulting in uterine enlargement, increased menstrual bleeding, prolonged menstruation, and progressively deteriorating dysmenorrhea. It is common among women of childbearing age. The reproductive tract microbiome of adenomyosis is currently the topic of many studies. A study analyzed the vaginal, cervical and uterine microbiota of patients with adenomyosis, patients with endometriosis, patients with both adenomyosis and endometriosis, and healthy women. It revealed that *Atopobium* was more abundant in patients with both adenomyosis and endometriosis simultaneously than in the other three groups (Chen et al., 2020). A cross-validated random forest model based on operational taxonomic units (OTUS) from the vagina, cervix, or uterus was developed to differentiate patients with and without adenomyosis (Chen et al., 2017). Studies with larger sample sizes have revealed that patients with adenomyosis had a higher alpha diversity of vaginal microbiota than the control group. In adenomyosis, the abundance of *Alloscardovia*, *Oscillospirales*, *Ruminoccoccaceae*, etc. increased, while the abundance of *Megaspehera*, *Fastidiosipila*, *Hungateiclostridiaceae*, and *Clostridia* decreased. While CST-III and IV were dominant in adenomyosis, CST-IV dominated the control group (Kunaseth et al., 2022). Another study focused on the endometrial microbiome and found that *Citrobacter freundii*, *Prevotellacopri*, and *Burkholderiacepacia* are the most promising microbial biomarkers for adenomyosis (Lin et al., 2023). The clinical application of these findings and the clarification of the adenomyosis pathogenesis require further study.

### 2.5 Endometrial hyperplasia

Endometrial hyperplasia, characterized by an increase in the number of dilated glands and the ratio of glands to stroma, is commonly diagnosed worldwide, which can coexist or gradually progress into endometrial cancer (Sanderson et al., 2017). Some patients with endometrial hyperplasia have irregular uterine bleeding, while others may not exhibit any symptoms at all and are only diagnosed during routine physical examinations. In terms of pathogenesis, this disease occurs by a microenvironment high in estrogen and a heightened inflammatory response (Kubyshkin et al., 2016). Atypical hyperplasia, a subtype of endometrial hyperplasia, is a premalignant lesion of endometrial carcinoma, receiving clinical attention. Because of the heterogeneity of endometrial hyperplasia, it is necessary to make a sensitive and accurate diagnosis of the actual premalignant lesions and the appropriate course of treatment are required. The disease must primarily be distinguished from normal proliferative endometrium and endometrial carcinoma. The main non-invasive auxiliary examination is an ultrasound; invasive surgery is required to collect the histopathology results to confirm the diagnosis. Clinically, ultrasonic measurement is significantly influenced by the technology and the operator, and it is more challenging to monitor in situations like obesity. The extent of endometrial hyperplasia is frequently restricted; hence, the ultrasound readings can occasionally be inaccurate. In clinical practice, it is easy to underestimate and overestimate the lesions. This clinical situation needs to be fixed. On the one hand, invasive procedures such as endometrial sampling or uterine curettage for women with simple hyperplasia or even normal proliferative endometrium is a type of excessive medical treatment, which not only results in a waste of medical resources but also the potential complications of these operations, such as uterine adhesion and infection, may harm these women, especially since the majority of these women are of childbearing age. On the other hand, misdiagnosing endometrial cancer as endometrial hyperplasia, or even misdiagnosing endometrial cancer and endometrial hyperplasia as normal endometrium, may result in errors in the selection of treatment and delay in the initiation of necessary treatment, which in the former case will prompt over-the-top medical care. Therefore, there is no doubt that the study of reproductive tract microbiota of endometrial hyperplasia is meaningful, which may help clarify the pathogenesis of endometrial hyperplasia and find suitable biomarkers for clinical diagnosis.

At present, most reproductive tract microbiome studies on endometrial hyperplasia studied endometrial carcinoma at the same time, and some studies even combined the two diseases into one group (Chao et al., 2022). A study published in 2016 included 4 patients with endometrial hyperplasia, 17 with endometrial carcinoma, and 10 with other benign gynecological diseases (Walther-António et al., 2016). The uterine α diversity in the hyperplasia group was similar to that in the carcinoma group, and was significantly higher than that in the benign group. Significant differences in the overall microbial flora structure among the three groups, mainly in the uterus rather than the vagina, were consistent with the α diversity analysis. The paired comparison of uterine samples showed no difference between endometrial hyperplasia and endometrial carcinoma. Simultaneously, there was a difference between the two groups and benign group respectively. According to the microbiome perspective, endometrial hyperplasia is the transitional stage from normal endometrium to endometrial cancer. The difference in the microbiota of endometrial hyperplasia from endometrial carcinoma and benign diseases makes exploring its characteristic microbial biomarkers possible. It is important to note that although correlation does not imply causation, the fact that two microorganisms found to be closely related to endometrial cancer, *A. vaginae* and *Porphyromonas* sp., did not exist in the vagina in endometrial hyperplasia, but exist in the uterine of half of the patients with endometrial hyperplasia, supported the role of these two microorganisms in early diseases, when taking into account the distance from the sample sites to the location of the disease. The primary shortcoming of this study is the insufficient number of patients with endometrial hyperplasia. Hence, the credibility of the relevant conclusions is low. Second, because the menopause and menstrual cycle were not considered in the design of the study, and because the factors mentioned above can affect the microbiome by hormone levels, it is crucial to evaluate the findings of this study carefully.

Another study included 26 patients with AUB and endometrial thickening identified by transvaginal ultrasound. Twelve of these patients had endometrial hyperplasia, and 14 had proliferative endometrium. In contrast to the previous study, it was discovered that the diversity of the vaginal microbial community in endometrial hyperplasia was much smaller than that in proliferative endometrium. This discrepancy may have resulted from differences in sample size, race, age, and other factors (Moosa et al., 2020). There were significant differences in the abundance of *Lactobacillus* in 15 genera, and if its abundance is used in the differential diagnosis of endometrial thickening, the sensitivity is 93%, and the specificity is 75% (Zhang et al., 2021). This study has a relatively sufficient sample size, and the choice of the control group is suitable for addressing clinical issues. Still, it ignores the impact of menstruation and menopause on the microbiome. According to the microbiome structure, endometrial hyperplasia can generally be distinguished from proliferative endometrium, suggesting that the microbiome may play a role in the development of the disease or that the difference may be a series of physiological changes in the local microenvironment brought on by the disease.

Even though the most recent study had a bigger sample size, it still mixed up pre-menopausal and post-menopausal patients, and its focus was only on the cervix and vagina (Barczyński et al., 2023). These research findings may aid in early detection and screening of endometrial hyperplasia using non-invasive techniques, given the accessibility of vaginal and cervical samples. However, the investigation of endometrial microbiota is more likely to further our understanding of pathogenesis. In general, we still know very little about the microbiome of endometrial hyperplasia and its subtypes, and the current research still has room to learn from the microbiome research of other diseases in terms of research design, as there are still a lot of open questions.

### 2.6 Endometrial cancer

Endometrial cancer is a common malignant tumor of the female reproductive system. Its pathogenesis has not been thoroughly elucidated, and genetics can only account for 20% of the incidence of endometrial cancer (Hampel et al., 2006). The remaining 80% of the causes could be related to hormones (Beral et al., 2005), diabetes (Friberg et al., 2007), and other potential pathogenic factors, and studies on these factors are intended to establish focused preventive interventions. It is proposed that dysbiosis may contribute to the activation of inflammatory pathway in endometrial cancer. Therefore, considerable research has investigated the interaction between the reproductive tract microbiota and endometrial cancer (**Table 1**).

**Table 1:** Studies in reproductive tract microbiota and endometrial cancer.

Reference	Year	Inclusion criteria	Exclusion criteria	Sample size for group A	Sample size for group B	Sample size for group C	Sample site	Methods	Findings
Walther-António et al. (2016)	2016	18 years or older; undergoing hysterectomy	pregnant or nursing; antibiotics within two weeks; using morcellation during the hysterectomy	10 (benign uterine conditions)	4 (endometrial hyperplasia)	17 (endometrial cancer)	Vaginal, cervical, uterine, fallopian, ovarian, peritoneal, and urine samples	16S rDNA V3-V5 region	*Firmicutes*, *Spirochaetes*, *Actinobacteria*, *Bacteroidetes*, and *Proteobacteria* are significantly enriched in endometrial cancer; the simultaneous presence of *Atopobium vaginae* and an uncultured representative of the *Porphyromonas* sp. (99 % match to *P. somerae*) is associated with endometrial cancer, especially if combined with a high vaginal pH (>4.5).
Walsh et al. (2019)	2019	total/subtotal hysterectomy	intravaginal infections within 3 months, allergy, autoimmune diseases, pregnancy, previous history of cancer	75 (benign uterine conditions)	7 (endometrial hyperplasia)	66 (endometrial cancer, 56 with type I, 10 with type II)	Vaginal, cervical, uterine, fallopian, ovarian, peritoneal, and urine samples	targeted qPCR	postmenopausal status is the main driver of a polymicrobial network associated with endometrial cancer; *Porphyromas somerae* is the most predictive microbial marker of endometrial cancer
Gonzalez-Bosquet et al. (2021)	2021	NA	NA	12 (normal)	112 (serous ovarian cancers)	62 (endometrioid endometrial cancers)	Tumor or normal fallopian tubes	Bacterial, archaea, and viral transcript (BAVT)	93 BAVTs differentially expressed between endometrioid endometrial cancer and serous ovarian cancer; endometrioid endometrial cancer BAVT expressions are between ovarian cancers and normal tubes
Lu et al. (2021)	2021	aged between 18 and 75 years, undergoing hysterectomy	pregnant or nursing, antibiotics or micro-ecologies within 3 months, infectious disease or genital tract medication within 3 months, preoperative chemotherapy or radiotherapy	25 (benign uterine conditions)	25 (endometrial cancer)	NA	Endometrial tissue	16S rRNA gene amplicon sequencing, real-time qPCR, Western blot.	*Micrococcus* was more abundant in the endometrial cancer, and *Pseudoramibacter_Eubacterium*, *Rhodobacter*, *Vogesella*, *Bilophila*, *Rheinheimera*, and *Megamonas* were less; rank correlation analysis showed a positive correlation between the relative abundance of *Micrococcus* and mRNA of IL-6 and IL-17
Li et al. (2021)	2021	aged between 40 and 69 years; undergoing hysterectomy	pregnant or nursing, antibiotics within three months, genital tract infection or medication within three months, preoperative chemotherapy or radiotherapy	10 (benign uterine conditions)	30 (stage I endometrial cancer)	NA	Endometrial tissue	16S rRNA sequencing	*Pelomonas* and *Prevotella* were more abundant in the endometrial cancer; the abundance of *Prevotella* and was positively corelated with serum D-dimer (DD) and fibrin degradation products (FDPs); transcriptome analysis identified 8 robust associations between *Prevotella* and fibrin degradation-related genes expressed within endometrial cancer; *Prevotella*, DD and FDPs showed a high potential to predict the onset of endometrial cancer (AUC = 0.86)
Hakimjavadi et al. (2022)	2022	18 years or older	neoadjuvant chemotherapy, douching within 14 days, vaginal cream or lubricant within 14 days, antibiotics within 14 days, or sexual intercourse within 5 days	11 (benign gynecologic disease)	30 (low-grade endometrial carcinoma)	20 (high-grade endometrial carcinoma)	vagina	shotgun metagenomic sequencing	*Fusobacterium nucleatum* was more abundant in group C than in group A; a significant increase in diversity from benign to HG disease; group A clustered in CST1, while group B clustered in CST2, and group C into both CST3 and CST4
Wang et al. (2022)	2022	Endometrioid adenocarcinoma; menopausal	current autoimmune diseases and gastrointestinal disorders, or a history of gastrointestinal surgery; genital tract infection and/or antimicrobial treatments to the genital area within 3 months; preoperative chemotherapy or radiotherapy; systemic antibiotics, corticosteroids, or any other immunosuppressive therapy within 6 months; smoking index >400; daily ethanol intake≥20 g in the past 5 years or ≥80 g in the past 2 weeks	28 (endometrial cancer)	NA	NA	Tumor tissue and adjacent non- endometrial cancer tissue	16S rRNA sequencing	The α diversity significantly increased in tumor tissues than those in adjacent non- endometrial cancer tissues. *Lactobacillus* and *Gardnerella* were the main bacterial genera in both tissues; *Prevotella*, *Atopobium*, *Anaerococcus*, *Dialister*, *Porphyromonas*, and *Peptoniphilus* were more abundant in the tumor tissue
Kaakoush et al. (2022)	2022	Postmenopausal; undergoing hysterectomy; body mass index (BMI) of either <27 kg/m^2^ (lean) or >30 kg/m^2^ (obese)	recipient of an investigational new drug within prior 6 days; antibiotics within 3 weeks	lean women with benign disease (n = 18), obese women with benign disease (n = 12)	lean women with endometrioid adenocarcinoma (n = 17), obese women with endometrioid adenocarcinoma (n = 23)	NA	endometrial tissues	16S rRNA transcript amplicon sequencing	obesity was not related to the type of microbial community in mouse endometrium in humans, the abundance of *Lactobacillus* in endometrial carcinoma was decreased, and obesity did not have association with it in mice, the abundance of *Lactobacillus* was positively correlated with normal uterine histology
Hawkins et al. (2022)	2022	Postmenopausal; Stage I endometrioid adenocarcinoma undergoing hysterectomy	preoperative chemotherapy or radiotherapy; antibiotics within 3 months	16 women in benign uterine conditions (2 Black, 13 White, 1 Other race).	95 women in the endometrioid adenocarcinoma group (23 Black, 72 White).	NA	Tumor tissues	16S rRNA sequencing	microbial diversity was decreased, and *Firmicutes*, *Cyanobacteria* and *OD1* were less in endometrial cancer from White versus Black women; *Dietzia* and *Geobacillus* was more abundant in tumors of obese White versus obese Black women
Barczyński et al. (2023)	2023	undergoing hysterectomy	intravaginal infection within 3 months, allergy, autoimmune diseases, pregnancy, previous history of cancer	27 (benign conditions)	21 (endometrial hyperplasia)	48 (endometrial cancer)	vaginal fornix and endocervical canal	Quantitative and qualitative real-time PCR analysis of DNA	*Lactobacillus iners* was significantly more frequent in group A, while *Dialister pneumosintes* and *Mobiluncus curtisii* were more frequent in cancer
Gonzalez-Bosquet et al. (2023)	2023	NA	NA	36 (normal)	112 (serous ovarian cancers)	62 (endometrioid endometrial cancers)	Tumor or normal endometrium	RNA sequencing and whole genome sequencing	*Desulfobacter*, *Desulfomicrobium*, *Parabacteroides*, and *Proteus* predicted endometrial cancer with AUC of 1.00; *Rhodopseudomonas* and *Proteus* had the most significant correlations with significant genes harboring SNVs
Byrd et al. (2023)	2023	NA	NA	528 (endometrial cancer)	NA	NA	NA	NA	The abundance of 11 common microbes changed with different MSI status in endometrial carcinoma, colorectal cancer and stomach adenocarcinoma

A paper published in 2016 found that *Firmicutes*, *Actinobacteria* and *Bacteroidetes* were more common in endometrial cancer than in benign uterine diseases. The sensitivity of combination of *A. vaginae* and the *Porphyromonas* sp. to diagnose endometrial carcinoma was 73%-93%, and the specificity was 67%-90% (Walther-António et al., 2016). Although there are a few drawbacks, it pioneers the way for future research. In a study released in 2019 that further examined how host variables influence the microbiome characteristics in endometrial cancer, and the post-menopausal state was found to be the primary driver of the network connected to endometrial cancer. When *P. somerae* was considered a predictive biomarker of endometrial cancer in all patients, the study found that the sensitivity of vaginal *P. somerae* was 74% and the specificity was 63%. In post-menopausal and obese patients, who were generally thought to be at high risk for endometrial cancer, the positive predictive value increased to 0.86. The study also focused on type I and type II endometrial cancers, two pathophysiological subtypes. Type I endometrial carcinoma, the endometrioid carcinoma, accounts for 70% to 80% of endometrial carcinoma, which frequently develops due to endometrial hyperplasia (Bansal et al., 2009). Type II typically has a *p53* gene mutation, which is more aggressive and has a worse prognosis. Early detection is crucial because Type II endometrial carcinoma sometimes lacks early symptoms like post-menopausal bleeding. Type I endometrial carcinoma is estrogen-dependent, while type II is not sensitive to estrogen. The study found that *P. somerae* was present in the vagina of all patients with type II endometrial cancer and 57% of patients with endometrial hyperplasia (Walsh et al., 2019), indicating that it is a suitable microbial marker for endometrial cancer. However, the use of *P. somerae* in diagnosing pre-menopausal and peri-menopausal endometrial cancer should be emphasized as constrained.

In a study of bacterial, archaea, and viral transcript (BAVT) in the tumor tissues of serous ovarian cancer and endometrioid endometrial cancer, and healthy fallopian tubes, the authors found that 93 BAVTs were expressed differentially in these three groups. Furthermore, the expression of 12 independently expressed BAVT in endometrioid endometrial cancer was higher than that in ovarian cancers and lower than that in healthy fallopian tubes. Potential targets for cancer therapy in the future may include the pathways connected to these BAVT (Gonzalez-Bosquet et al., 2021). Further study found that microbial communities of the uterus correlated with genetic variation (Gonzalez-Bosquet et al., 2023). The study validated these results in separate datasets, the Cancer Genome Atlas (TCGA) and Gene Expression Omnibus databases, strengthening the reliability and universality of the research conclusions. However, it did not control confounding variables like age and menopause. Another study explored the relationship between endometrial microbiota, the expression of inflammatory cytokines, mRNA and proteins of interleukin (IL)-6, IL-8 and IL-17, and disease state. It was noted that there were notable differences in 12 genera between the endometrial cancer group and the benign uterine disease control group. *Micrococcus* was more abundant in the endometrial cancer group, while other genera were less in the endometrial cancer group. In terms of inflammatory factors, this study found that endometrial cancer had higher mRNA expression of IL-6, IL-8 and IL-17, and higher levels of IL-6 protein than control. Rank correlation analysis showed a positive correlation between the relative abundance of *Micrococcus* and mRNA of IL-6 and IL-17 (Lu et al., 2021). This study has a substantial number of samples, but unfortunately the menopausal status of patients was not adequately considered in the study design, and there was a statistical difference in age between the endometrial cancer group and the control group. The range of applications for the results must be determined by additional research. Another article published in 2021 examined for the first time the correlation between endometrial microbiota and tumor transcriptome in endometrial cancer and the relation between endometrial microbiota and blood coagulation indicators. *Pelomonas* and *Prevotella* were more abundant in the endometrial cancer than the benign uterine disease control. Further study showed that *Prevotella* abundance was positively correlated with serum D-dimer (DD) and fibrin degradation products (FDPs). The microorganism-related transcriptome of tumor tissue provided an explanation for the correlation. *Prevotella*, combined with DD and FDPs, had excellent potential for predicting endometrial cancer existence (Li et al., 2021). It should be carefully considered whether this conclusion may be generalized to younger pre-menopausal and peri-menopausal patients with endometrial cancer because the patients included in this study were relatively old.

A study focused on the vaginal microbiota at various endometrial cancer malignancy levels. It reported that *Fusobacterium nucleatum* was more abundant in high-grade endometrial carcinoma than in benign gynecologic disease. The diversity increased greatly from benign to high-grade endometrial carcinoma and the benign condition, low-grade endometrial carcinoma and high-grade clustered into CST1, CST 2, and both CST3 and CST4, respectively (Hakimjavadi et al., 2022). The limitation of this study is that there were significant differences across groups in several clinical and demographic characteristics, including age and ethnicity. Another article in 2022 focused on postmenopausal endometrioid adenocarcinoma. It compared and analyzed the microbiota of tumor tissue and paracancerous tissue, and discovered that the α diversity and evenness of tumor tissue were significantly higher than those of paracancerous tissue, and *Prevotella*, *Atopobium*, and *Porphyromonas* were more abundant in the tumor tissue (Wang et al., 2022).

Lean and obese mice models were utilized to test the hypothesis that the endometrial microbiota is associated with obesity and endometrial cancer. It was found that obesity was unrelated to the type of microbial community in mouse endometrium. In humans, the abundance of *Lactobacillus* in the endometrium of postmenopausal patients with endometrial carcinoma was decreased compared with patients without endometrial carcinoma, but obesity did not have association with its abundance. In mice, the abundance of *Lactobacillus* was positively correlated with healthy uterine histology (Kaakoush et al., 2022). These findings suggested that *Lactobacillus* may preserve the endometrium, whereas endometrial carcinoma and obesity may impact the microorganisms living within the endometrium. Another study looked into how race as a demographic characteristic affected the microbiota. In contrast to black women, white women had lower *Firmicutes*, *Cyanobacteria*, and OD1 levels in their endometrial cancers, according to this study (Hawkins et al., 2022). TCGA database was used creatively in this study, which future researchers should note. A study based on TCGA database found that the abundance of 11 common microbes changed with different microsatellite instability (MSI) status in endometrial carcinoma, colorectal cancer and stomach adenocarcinoma (Byrd et al., 2023) which indicated that the intratumor microbiota may be different with various MSI status and affect the tumor microenvironment.

Despite the availability of numerous technological techniques, there is still a lack of research on premenopausal endometrial cancer. It is important to note that in clinical practice, the pathology of endometrial biopsy or curettage may be inconsistent with the pathology of hysterectomy, which makes it challenging for a portion of young patients to choose the appropriate therapy, as they may desire to preserve reproductive function. In the present, they can only rely on inaccurate biopsy pathological results when making treatment choices. Therefore, if we can figure out suitable microbial biomarkers to assist in determining determinthe pathological subtype of premenopausal endometrial carcinoma, whether to retain reproductive function or not will be decided with more certainty.

## 3 Discussion

**Figure 1:**
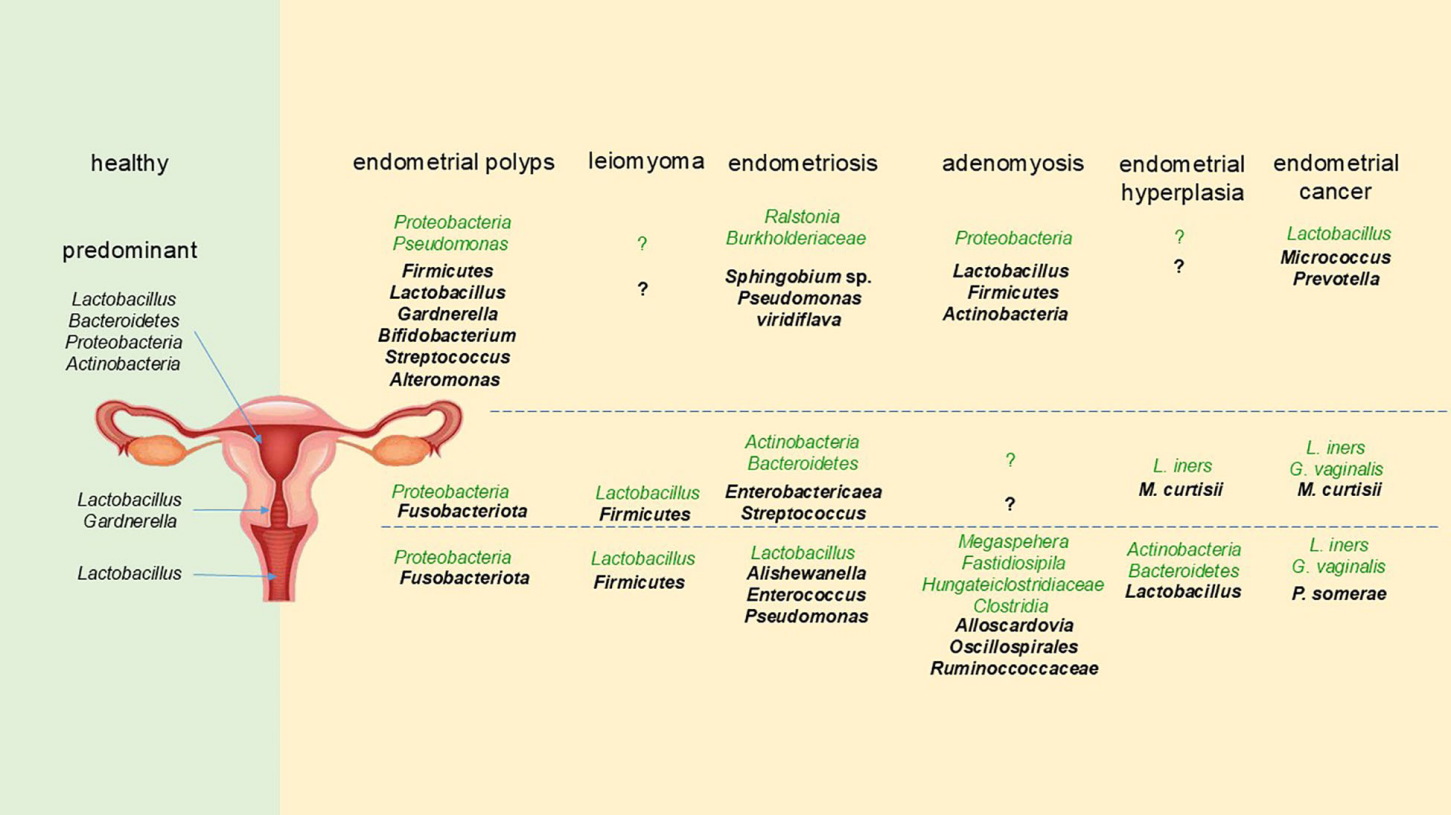
The changes of female reproductive tract microbiota in different gynecological diseases. The format of the font represents different trends compared with healthy women, with the bold and darker fonts representing an increase, and the normal and lighter fonts representing a decrease.

We summarized findings of variations in the microbiota of the female reproductive tract between different disease states and healthy women (**Figure 1**). Comparing the microbiota of the same disease in several regions of the reproductive tract longitudinally, we found that when there was a change in one microorganism, its trend in the entire reproductive tract was frequently the same. For instance, in endometrial polyps, the level of *Proteobacteria* in the entire reproductive tract was lower than that in healthy women, and *Lactobacillus* showed a decreasing trend throughout the entire reproductive tract of endometrial carcinoma. Not surprisingly, there were some differences in the changes of microbiota in different parts of the reproductive tract of the same disease, just as there are varied flora composition patterns in diverse locations in healthy women (Chen et al., 2017). By comparing various diseases horizontally, we found that the increase or decrease of *Lactobacillus* was related to all the diseases we reviewed. Specifically, the decrease of vaginal *Lactobacillus* was associated with leiomyoma (Chen et al., 2017), endometriosis (Chao et al., 2021; Lu et al., 2022), and endometrial cancer (Kaakoush et al., 2022). The increase in the abundance of *Lactobacillus* in the reproductive tract was associated with endometrial polyps (Fang et al., 2016), adenomyosis (Lin et al., 2023), and endometrial hyperplasia (Zhang et al., 2021). This demonstrated that *Lactobacillus*, one of the dominating phyla in all regions of the female reproductive tract, was closely related to the health status of women. If its abundance changes noticeably, it is important to be vigilant about the gynecological diseases. Besides, *Firmicutes* (Fang et al., 2016; Hawkins et al., 2022; Hua et al., 2022; Kaakoush et al., 2022; Liang et al., 2023; Medina-Bastidas et al., 2022) and *Streptococcus* (Gonzalez-Bosquet et al., 2021; Kaakoush et al., 2022; Lee et al., 2021; Liang et al., 2023) always increased in abundance when women experienced various gynecological conditions, suggesting that these bacteria can be harmful to the health of women. If an increase in these bacteria is detected, it is important to be aware of the potential diseases.

There is some basic research concerning the mechanisms of genital microbiome in gynecological diseases. Under normal conditions, the vagina maintains an acidic pH (3.0–4.5), which is primarily due to the secretion of lactic acid and hydrogen peroxide by *Lactobacillus* species. *Lactobacillus* species not only produce bacteriocins, which, with the acidic pH, discourage the growth of pathogens, but they also adhere to epithelial surfaces, preventing the adhesion of pathogenic microorganisms (O’Hanlon et al., 2011; Ravel et al., 2011). They also promote autophagy of infected cells, eliminating viruses, bacteria, and protozoa (Ghadimi et al., 2010), and modulate inflammatory responses, particularly during pregnancy (Aldunate et al., 2015). Loss of *Lactobacillus* dominance, coupled with an increase in microbial diversity, is often associated with immune and epithelial homeostasis alterations. This may be induced by a series of mechanisms, such as: (a) the production of pro-inflammatory cytokines and chemokines, (b) the recruitment of immune cells, and (c) a reduction in the viscosity of cervicovaginal fluid due to the activity of mucin-degrading enzymes, including sialidase, fucosidase, galactosidase, N-acetyl-glucosaminidase, and various aminopeptidases (Moncla et al., 2015; Olmsted et al., 2003). Dysbiosis may also have far-reaching effects on immune and metabolic signaling, potentially influencing the development of gynecological diseases such as cervical, endometrial, and ovarian cancers. These pathophysiological changes can contribute to chronic inflammation, epithelial barrier dysfunction, alterations in cell growth and apoptosis, genomic instability, angiogenesis, and metabolic dysfunction (Wu et al., 2015). Emerging evidence suggests that reproductive organ dysfunction, in conjunction with specific bacteria, could play an active role in the development, progression, and metastasis of gynecologic malignancies, possibly through mechanisms such as the regulation of estrogen metabolism (Łaniewski et al., 2020). In benign gynecological diseases, studies in non-human primates (*Papio anubis*) have been conducted. One study found that induction of endometriosis in primates resulted in a systemic inflammatory response, characterized by a decrease in peripheral Tregs and an increase in Th17 cells, both of which are markers of systemic inflammation. Following the induction of endometriosis, the diversity and abundance of the microbiome in the vagina were altered, suggesting that shifts in the mucosal microbial community may contribute to ongoing inflammation by producing inflammatory mediators (Le et al., 2022).

Because of the tremendous diversity of the microbiota and the enormous variances across patients, an effective analytical method is required to describe the overall alterations in the microbiota more concisely. The application of CST filled in the gaps by summarizing the characteristics of the microbiota. This technique categorizes microorganisms, which is convenient for researchers to analyze the broad aspects of changes in microbiota (Brooks et al., 2017; Ravel et al., 2011).

There have generally been few statistically significant findings about endometrial microbiota in various gynecological diseases, which can be ascribed to several factors. First, the difficulties of sampling may have prevented adequate meaningful exploration. In addition, some studies conducted the research with only a few positive findings revealed, which was potentially caused by the low microbial biomass in the uterine cavity. As a consequence, the results were easily affected by sample contamination. Moreover, low abundance made sequencing more challenging (Molina et al., 2021). Therefore, future research should pay attention to standardizing sampling, preservation, extraction, and sequencing procedures, and strictly controlling contamination at every step of the operation process to acquire statistically significant results reflecting the changes in endometrial microbiota.

This paper reviewed the most recent research on the reproductive tract microbiota in common benign and malignant gynecological diseases, including endometrial polyps, uterine fibroids, endometriosis, adenomyosis, endometrial hyperplasia, and endometrial carcinoma. Numerous studies have demonstrated that the microbiota of the female reproductive tract is different in different health states. Additionally, specific bacteria may promote the development of the certain diseases, and the therapy targeting microbiota showed certain efficacy in the experiment. Simultaneously, it is acknowledged that we do not thoroughly comprehend the mechanism of interaction between the human body and the microbiota of the reproductive tract.

An examination of the literature revealed that, despite the relatively advanced technology, the knowledge of microbiota in various diseases varied greatly in different diseases. For instance, there are more studies on endometriosis and endometrial carcinoma than adenomyosis and endometrial hyperplasia, some of which have even explored the subtypes of the diseases (Yang et al., 2023). The discrepancy in disease incidence and the level of clinical concern of diseases could cause the variance. Since endometriosis is more common than adenomyosis, finding patients who match the inclusion requirements is simpler. Additionally, other factors can make it more challenging to collect enough samples. For instance, women of childbearing age comprise most adenomyosis patients. Gathering enough uterine samples for research is difficult because only a small portion of them chooses hysterectomy. Some of the issues can be resolved by inter-institutional collaboration, and patients without surgical plans may be subjected to alternate sampling techniques, including endometrial sampling brushes for uterine microbiota.

Some studies describe and analyze the sampling results of reproductive tract microbiome in common gynecological diseases, but these results can only prove the correlation. There are few studies on the interaction between microbiota and disease state. Future research can make use of several established techniques, including simultaneously examining the transcriptome and proteome (Li et al., 2021), exploring the pathways and mechanisms underlying the interaction between the human body and the microbiota using primary cultured tissues *in vitro* (Guo et al., 2015), and utilizing animal models to investigate the causal relationship between alterations in microbiota and the onset of disease (Lu et al., 2022). Lastly, return to the clinic and conduct clinical trials of treatments targeting microbiota (Khan et al., 2018). Numerous studies and efforts are required to identify significance and clarify the possible mechanism.

The research of reproductive tract microbiome may contribute to resolving several clinical issues, including the causes of endometrial polyps recurrence, which patients with endometrial hyperplasia are more likely to develop endometrial cancer, and the considerations that should be made when young patients with endometrial cancer opt for fertility-preserving treatment. In large clinical studies, particularly in long-term follow-up cohort studies and randomized controlled trials, the inclusion of reproductive tract microbiome research will help to acquire more comprehensive and detailed results.

In the future, more high-quality microbiome studies will provide information for the prevention, screening, diagnosis and treatment of common gynecological diseases. In particular, because the endometrial microbiome is frequently closer to the site of the lesion and is more likely to directly interact with the disease, it is more likely to be significant for the study of the pathogenesis of the disease, which may help inform the development of prevention and treatment strategies. Because of the convenience of vaginal sampling, the results of vaginal microbiome studies are more suitable for disease screening and diagnosis. The cervix locates halfway between the two sites, combining both the advantages and disadvantages. We should base our choice of research location on clinical problems we hope to resolve.

After being properly validated, it is anticipated that treatments aimed at the microbiome, such as antibiotics, probiotics, and flora transplantation, will be exploited in clinical practice to alleviate the suffering of patients.

## 4 Conclusion

The study of human reproductive tract microbiota has been a rapidly developing and promising field recently. It has been demonstrated that in different gynecological disease states, the female reproductive tract microbiota is different. Additionally, some bacteria may induce the disease progress, and therapies targeted microbiota, such as antibiotics, probiotics, and flora transplantation, have been successful in the trial. These findings highlight the significance of symbiotic microbiota in the reproductive tract in preserving health. By merging other technologies, such as transcriptome and proteome, *in vitro* cultured cells, and animal models, we can comprehend how reproductive tract microbes interact with their hosts to protect health or cause disease. The information obtained from more high-quality microbiome studies can contribute to screening and diagnosis of gynecological diseases as well as the development of preventative and therapeutic strategies aimed at preserving or reestablishing healthy reproductive tract microbiota.
